# Identification of *ISCA1* as novel immunological and prognostic biomarker for bladder cancer

**DOI:** 10.3389/fimmu.2022.975503

**Published:** 2022-08-22

**Authors:** Renlong Zhou, Naixiong Peng, Wei Li

**Affiliations:** ^1^ Department of Blood Transfusion, Shenzhen Longhua District Central Hospital, Shenzhen, China; ^2^ Department of Urology, Shenzhen Longhua District Central Hospital, Shenzhen, China

**Keywords:** pan-cancer, *ISCA1*, BLCA, GSEA, immune microenvironment, prognostic analysis

## Abstract

**Background:**

Iron-sulfur cluster assembly 1 (*ISCA1*) has a significant effect on respiratory complexes and energy metabolism. Although there is some evidence that *ISCA1* gene expression impacts energy metabolism and consequently has a role in tumorigenesis and cancer metastasis in different types of malignancies, no systematic pan-cancer study of the *ISCA1* has been conducted. As a result, we sought to investigate *ISCA1*’s predictive value in 33 cancer types as well as its possible immunological function.

**Methods:**

We included the pan-cancer expression profile dataset and clinical data from the public database. Firstly, the single-sample Gene Set Enrichment Analysis (ssGSEa) approach was employed for analyzing the immune link in pan-cancer, while the limma package was utilized for analyzing the differential expression in cancer species. Subsequently, ciberport, MCP-counter, TIMER2, quanTIseq, and xCELL were employed for analyzing bladder cancer (BLCA)’s immune infiltration. Least absolute shrinkage and selection operator (Lasso) were employed for choosing the best gene to develop the immune risk scoring model.

**Results:**

*ISCA1* gene expression was positively related to four immune signatures (chemokine, immunostimulator, MHC, and receptor) in BLCA. Samples of BLCA were sorted into two groups by the best cut-off of *ISCA1* expression degree. The group with a high level of *ISCA1* expression had a higher risk, suggesting that the ISC*A1* gene was a risk factor in BLCA, and its high expression resulted in a poorer prognosis. Additionally, it was noted that *ISCA1* was positively linked with these immune checkpoints. Moreover, there was a considerable positive link between *ISCA1* and different immune properties in subgroups with different immune checkpoint inhibiting responses. Finally, an immune risk scoring model was made and it showed a better score in comparison to that of TIDE.

**Conclusion:**

*ISCA1* can be a prognostic marker for a variety of cancers, particularly BLCA. Its high level of expression has a deleterious impact on the prognosis of BLCA patients. This strongly shows that *ISCA1* is a significant prognostic factor for BLCA and that it could be used as a new prognostic detection target and treatment approach.

## Introduction

Cancer is the primary cause of mortality and a major setback to improving the quality of life all over the globe. There is no ultimate treatment for it as of the present day (1). Recently, cancer immunotherapy, particularly immune checkpoint blocking therapy has become a prominent cancer treatment approach (2). New immunotherapy targets can be found through pan-cancer expression analysis of genes and examination of their linkage with clinical prognosis and the associated signal pathways, thanks to the ongoing development and improvement of public databases like The Cancer Genome Atlas (TCGA) (3).

Mitochondria have become important pharmacological targets due to their essential role in cellular growth and apoptosis (4). Mitochondria in tumor tissues can transform metabolic phenotypes to cope with the high energy demand and macromolecule synthesis (5, 6). Additionally, mitochondria can interact with the tumor microenvironment, and signals from fibroblasts related to cancer have an impact on them (7). *ISCA1* variant has been linked to mitochondrial malfunction (8), mainly because *ISCA1* regulates the expression of essential proteins in the mitochondrial respiratory chain complex, having a significant impact on it as well as energy metabolism. *ISCA1* is an evolutionarily conserved type A ISC protein involved in Fe-S synthesis. Knockdown investigations in HeLa cells of two type A proteins, *ISCA1* and ISCA2, reveal that these two proteins may have a function in the increased synthesis of mitochondrial Fe4S4 in humans (9). Using recombinant human *ISCA1* and ISCA2, recent *in vitro* biochemical experiments have confirmed cluster transfer and protein-protein interaction between human glutaredoxin GLRX5 and *ISCA1* or *ISCA2* (10).

Although, some research has been done on the role of *ISCA1* in malignancies. Only relevant research has revealed that Integrin Subunit Beta 3 (*ITGB3*) affects energy metabolism through the expression of the *ISCA1* gene, which has a role in breast cancer bone metastases (11). As a result, the possible role of *ISCA1* in a range of malignancies has to be investigated in detail. The expression level of *ISCA1* in different forms of cancer and its connection with prognosis were studied using two databases: TCGA and Gene Expression Omnibus (GEO). It also discussed the association between *ISCA1* expression and immunity in 33 tumors. Following that, it was discovered that bladder cancer (BLCA) had the strongest link to immunity. The researchers next looked into the possible links between *ISCA1* and mutation analysis, DNA methylation, tumor mutational burden (TMB), immunological infiltration, and clinical response. In addition, the biological function of *ISCA1* in malignancies was investigated using protein-protein interaction (PPI) analyses between immune-linked differential genes and *ISCA1*. Finally, the immunological risk score (IRS) model was developed, and its result was superior to the TIDE result. Finally, our findings indicated that *ISCA1* could be a predictive factor for bladder cancer (BLCA). *ISCA1* may alter tumor-infiltrating immune cells, which could be majorly involved in tumor immunity. This research could help researchers better grasp ISCA1’s involvement in tumor immunotherapy.

## Methods

### Data source and pretreatment

The RNA sequencing (RNA-seq) expression profile data, somatic mutation data, and survival data regarding pan-cancer (33 species) were taken from the database of UCSC Xena (https://xenabrowser.net/). The format of RNA-seq data was changed from Fragments Per Kilobase Million (FPKM) to the format of Transcripts per million (TPM), and then we did a log2 conversion. Among them, analysis and processing of the downloaded somatic mutation data were done by mutect. Finally, the copy number variations (CNV) data processed by the gistic algorithm was also provided by the UCSC Xena database (http://xena.ucsc.edu/), while the methylation data was taken from the LinkedOmics database (http://linkedomics.org).

The BLCA GEO queue was retrieved from the GEO database (https://www.ncbi.nlm.nih.gov/geo/), which has extensive survival data, such as GSE31684, GSE48075, GSE13507, GSE32894, GSE48277, and GSE69795. The BLCA samples were kept.

Afterward, we also downloaded three cohorts linked with immunotherapy, GSE78220 (melanoma), GSE135222 (NSCLC), and GSE91061 (melanoma). Following the knowledge sharing 3.0 License Agreement, the complete expression data and comprehensive clinical data of the IMvigor210 queue (BLCA immunotherapy-related queue) came from http://research-pub.Gene.com/imvigor210corebiologies/.

### Analysis of tumor immune microenvironment and immune infiltration

#### Single sample gene set enrichment analysis

Single sample gene set enrichment analysis (ssGSEA) (12) was proposed for the first time in 2009 and was made for a single sample that could not be utilized for GSEA. The R package GSVA can be used to implement it. At present, ssGSVA is frequently utilized for assessing the extent of tumor immune cell infiltration.

##### Estimate

Moreover, we utilized estimate (13) for evaluating the tumor immune microenvironment scores of samples, and then a comparison of their differential distribution in different subtypes was done. Following the expression data, estimate provided scholars with tumor purity scores, the stromal cells’ level, and the immune cell infiltration level in tumor tissue.

##### Ciberport

Deep learning algorithms such as convolution and deconvolution are commonly known. Each sample is treated as a mixture of numerous immune cells in this procedure. The link between the components and expression of each immune cell and the final combination is fit using linear regression. The expression properties of each immune cell were retrieved using a deconvolution technique. The method of calculating immune cell infiltration known as CIBERPORT (14) is widely utilized. For estimating the abundance of immune cells, it employs the technique of linear support vector regression to deconvolute the expression matrix of immune cell subtypes.

#### Tumor immune estimation resource

The Tumor Immune Estimation Resource (timer) is one of the procedures for deconvolution of cell mixtures following the expression characteristics (15). Timer2 is one of the most widely utilized approaches for immune infiltration analysis in bioinformatics. MCP-counter (Microenvironment Cell Populations-counter) (16) is an R tool that uses normalized transcriptome data to quantify the absolute abundance of eight immune cells and two stromal cells in diverse tissues. The score can be used to demonstrate the degree of infiltration in the immunological milieu, but the number of cells cannot be compared. ESTIMATE can’t assess particular immune cell infiltration; it can only assess immune cell purity, tumor cell abundance, and stromal cell abundance.

#### QuanTIseq

The QuanTIseq (17) was utilized for quantifying both the tumor immune status according to the human RNA-seq data as well as the proportion of ten distinct types of immune cells along with other non-characterized cells in the sample by deconvolution.

#### Xcell

Xcell (18) is an ssGSEA-based procedure with the ability to do cell type enrichment analysis according to gene expression data of 64 types of immune and stromal cells. Since the Xcell employs expression level ranking rather than the actual value, normalization has no effect, although the input data requires a normalization format. As a result, the immune infiltration of BLCA was analyzed using CIBERPORT, MCP-counter, TIMER2, quanTIseq, and Xcell, and the connection between the expression of *ISCA1* and their scores were measured.

### GSEA and annotation of differentially expressed genes

The analysis difference between subtypes was done using the limma package (19), and differentially expressed genes were chosen through the | log2 (Fold Change) | >1 and False Discovery Rate (FDR) <0.05.

We enriched the differentially expressed genes among subtypes and then carried out an analysis using Gene Ontology (GO) and Kyoto Encyclopedia of Genes and Genomes (KEGG) through the WebGestaltR package (version 0.4.2) (20), and the chosen gene set was “c2 cp. kegg. v7.0. symbols. Gmt”, which had the KEGG channel. The GSEA input file consisted of the expression profile data. The threshold values of enriched pathways were p<0.05 and FDR < 0.25. Likewise, the GO function enrichment analysis of differentially expressed genes was done using the R software package WebGestaltR (threshold value was set as P < 0.05).

### Univariate and multivariate cox analysis

The R-package survminer (https://cran.r-project.org/package=survminer) was employed to get the best cutoff of genes from various datasets, and the samples were sorted into high and low expression groups following the best cutoff, and afterward, we drew the KM curve. We randomly collected the BLCA cancer samples from the TCGA dataset using the ratio train:test = 7:3. Univariate analysis was done in the training data set. The R software package glmnet (21) was utilized for establishing the Lasso expression model (COX). Based on the model created in this study, the most suitable genes were chosen using single factor Cox regression, and we obtained 21 genes when the value of minimum lambda=0.04090851, which were employed for multivariate analysis. StepAIC approach was utilized for reducing gene number. The stepwise regression used the Akaike information criterion (AIC) (22), which took into account the model’s statistical fit and the number of parameters used for fitting. The stepAIC procedure in the MASS package started from the most complex model and successively eliminated a variable to lower the AIC. The smaller the value, the better the model, which suggested that the model had a sufficient fit with fewer parameters. 11 genes were obtained at the end according to this procedure.

### TIDE analysis of immunotherapy effect

Through a comprehensive study of hundreds of various tumor expression profiles, the Tumor Immune Dysfunction and Exclusion (TIDE) (http://TIDE.dfci.harvard.edu/) (23) analysis can uncover biomarkers that predict the therapeutic response of immune checkpoint inhibitors/medicines. The TIDE score obtained from TIDE analysis can be used to determine the sensitivity of immunological checkpoints.

### Tumor mutation burden

TMB is a quantifiable immune-response biomarker that reflects the number of mutations in tumor cells. TMB scores were calculated using a Perl script and corrected by dividing by the total length of exons.

### ssGSEA

Base on genes from previous research (24) and ssGSEA analysis was used to analyze these genes to define T cell inflamed score.

### Statistical analysis

The difference in clinicopathological features among the three subtypes was investigated using the Chi-square test. The expression levels of three subtypes were determined using ANOVA. The difference in the two groups was investigated using the T-test. For correlation analysis, the Pearson correlation coefficient was used. R (version 4.0.2) was used for statistical analyses. Statistical significance was defined as a P-value of < 0.05.

## Results

### Immune correlation of *ISCA1* gene in pan-cancer

**Figure 1:**
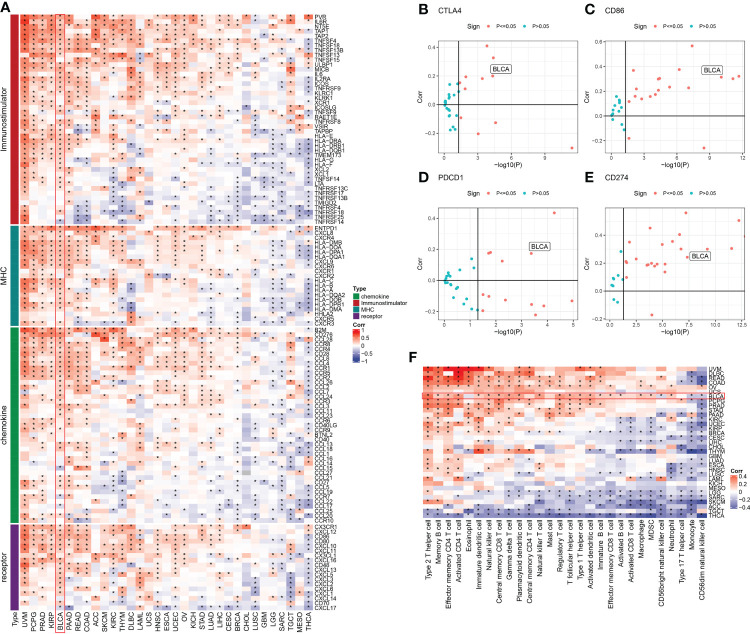
Immune correlation analysis of *ISCA1* gene in pan-cancer. **(A)** The link between *ISCA1* and immunomodulators (chemokines, receptors, MHC, and immune stimulants). **(B–E)** The links between *ISCA1* and four immune checkpoints (PDCD1, CTLA4, CD274, and LAG3), which represent the type of cancer, was examined. The Y-axis indicated Pearson correlation and the X-axis indicated -log10 (P-value). **(F)** Spearman correlation analysis was used to measure the link between the expression of the *ISCA1* gene in 33 different types of cancer and 28 tumor-related immune cells. The color represents the correlation coefficient. An asterisk indicates a statistically significant P-value calculated using Spearman correlation analysis. (* stands for p<0.05).

We discovered four types of genes in the literature including MHC, chemokine, immune-stimulator, and receptor (25). The Spearman correlation between these genes and the *ISCA1* was measured in pan-cancer. The link between these four gene types with *ISCA1* was varied in various types of cancer. It was mostly positive in uveal melanoma (UVM), BLCA, kidney papillary cell carcinoma (KIRP), etc. while thyroid carcinoma (THCA), testicular germ cell tumors (TGCT), etc. were mostly linked negatively (**Figure 1A**). Moreover, the link between CTLA4, PDCD1, CD86, CD274, and *ISCA1* in various cancer types was measured. The outcomes revealed that these four genes were substantially positively linked with *ISCA1* in BLCA (**Figures 1B–E**). Furthermore, ssGSEA was employed for evaluating the scores of 28 immune cell scores in various cancer types, and then their link with *ISCA1* was measured. The outcomes of this analysis suggested that there was a considerable positive link between the expression of *ISCA1* and 20 immune scores in BLCA (**Figure 1F**).

**Figure 2:**
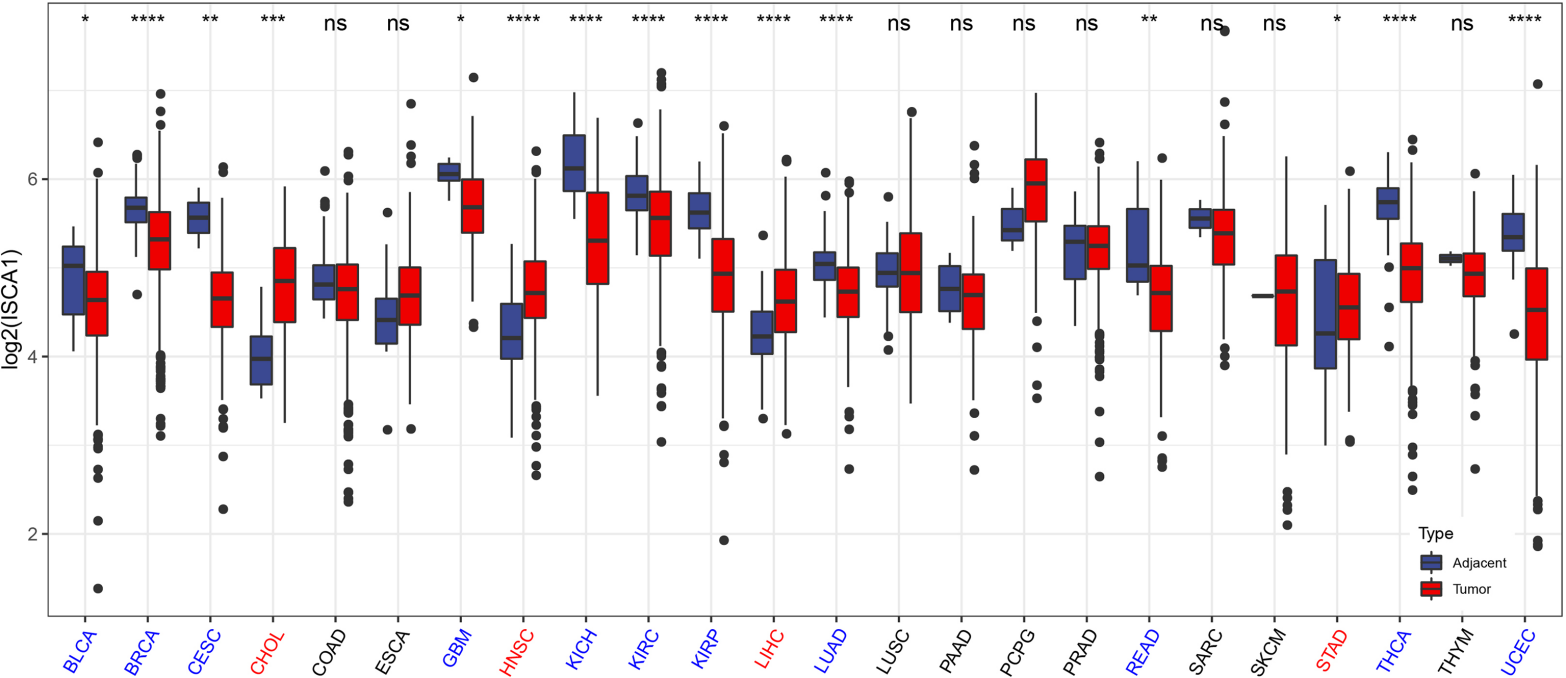
The expression of the *ISCA1* gene in pan-cancer is varied from that in adjacent cancer. Blue represents significantly low expression in tumor, and red represents significantly high expression in tumor (* represents p < 0.05, ** represents p < 0.01, *** represents p < 0.001, **** represents p < 0.0001, ns represents p > 0.05).

Moreover, the *ISCA1* gene expression in pan-cancer was observed (**Figure 2**). The outcomes indicated that: among the 24 tumors with para-cancerous samples, the expression of the *ISCA1* gene in 15 cancer species was considerably varied in comparison to that in para-cancerous samples. Among them, the expression of the *ISCA1* gene was lowered in tumor samples of 11 cancer species, including breast invasive carcinoma (BRCA), BLCA, cervical squamous cell carcinoma (CSCC), glioblastoma multiform (GBM), kidney chromophobe (KICH), endocervical adenocarcinoma (CESC), kidney renal clear cell carcinoma (KIRC), KIRP, THCA, endometrial carcinoma (UCEC), lung adenocarcinoma (LUAD), rectum adenocarcinoma (READ), etc. Moreover, the expression of cholangiocarcinoma (CHOL), head and neck squamous cell carcinoma (HNSC), liver cancer (LIHC), and stomach cancer (STAD) were enhanced in tumor samples of four cancer species.

### SNV, CNV, and methylation analysis in BLCA

Based on the above analysis of pan-cancer, the *ISCA1* gene had a considerable positive link with four types of genes in BLCA: immune-stimulator, MHC, chemokine, and receptor. In BLCA, the *ISCA1* gene was positively linked with CTLA4, PDCD1, CD86, CD274, and immune score. Using the difference analysis, it was discovered that the expression of the *ISCA1* gene in BLCA tumor samples was decreased in comparison to that in adjacent samples. Furthermore, the survival analysis highlighted that samples were divided into the high *ISCA1* expression group and low *ISCA1* expression group., so we were focused on the role of *ISCA1* in BLCA.

**Figure 3:**
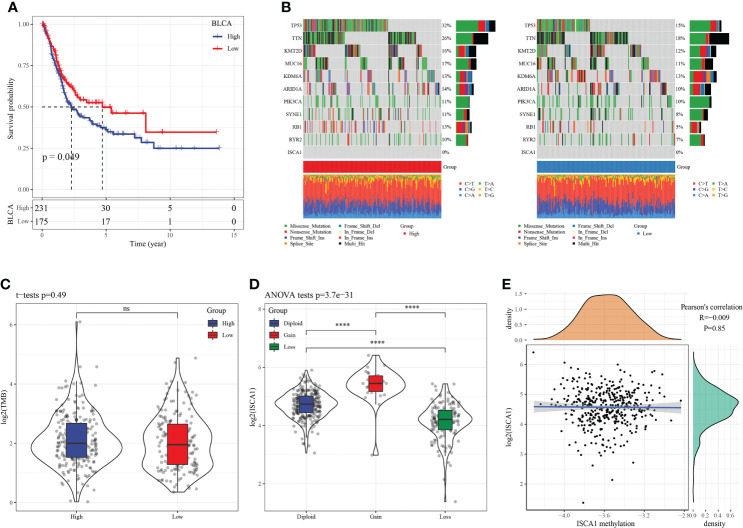
SNV, CNV, and methylation analysis in BLCA. **(A)**: In BLCA, the KM curve showed that patients in high *ISCA1* group had worse survival outcome compare to low *ISCA1* group, both of which were borderline significant; **(B)**: The mutation distribution of the top 10 genes with the highest frequency of mutations in the high *ISCA1* expression group and low *ISCA1* expression group; **(C)**: The distribution of TMB in high *ISCA1* expression groups and low *ISCA1* expression group was compared; **(D)**: The gene expression difference of *ISCA1* in *ISCA1* gene amplification group; **(E)**: Correlation analysis between gene *ISCA1* expression and methylation (**** represents p<0.0001, ns represents p > 0.05).

In herein, we used the surv_cutpoint function of the SurvMiner package to find the best cutoff for grouping. BLCA samples were categorized into two groups (**Figure 3A**) as per the best cutoff of *ISCA1* expression value. The group with high *ISCA1* expression showed a poor prognosis, suggesting that *ISCA1* is a risk factor in BLCA. Afterward, we mapped 10 genes with the highest mutation frequencies in the high and low expression groups. The outcomes revealed that TTN, synb1, TP53, RB1, kmt2d, arid1a, and other genes in the low expression group had lower mutation frequencies (**Figure 3B**). However, no major variation was observed in the TMB of high and low expression groups of *ISCA1* (**Figure 3C**). We observed that there was a major difference in the expression of *ISCA1* with CNV amplification and deletion and the normal copy number, and the expression of *ISCA1* with CNV amplification was greatly enhanced, while that with CNV deletion was greatly reduced (p < 0.0001, **Figure 3D**). Meanwhile, no major link was seen between the methylation degree and the expression of *ISCA1* (**Figure 3E**). Also, using limma analysis, 1672 genes, included 1505 upregulated genes and 167 downregulated genes, were screened between high ISCA1 group and low ISCA1 group (**Figure S1A**). KEGG pathways enrichments analysis showed that 1505 genes were enriched in 10 KEGG pathways, such as, PI3K-Akt signaling pathway, cell cycle (**Figure S1B**), while 167 genes were not enriched into the KEGG pathway.

### Comparative analysis of the immune status of *ISCA1* groups in BLCA

**Figure 4:**
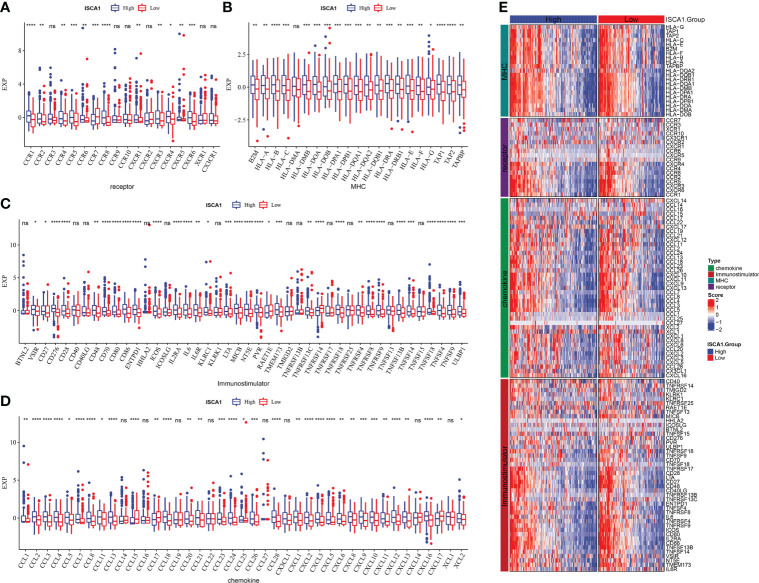
Comparative analysis of the immune status of *ISCA1* group in BLCA. **(A–D)**: The expression differences of different types of genes (chemokines, receptors, MHC, and immunostimulants) grouped by *ISCA1* in BLCA; **(E)** Heatmap of differences in the expression of different types of genes (chemokines, receptors, MHC, and immunostimulants) grouped by *ISCA1* in BLCA (* represents p < 0.05, ** represents p < 0.01, *** represents p < 0.001, **** represents p < 0.0001, ns represents p > 0.05).

In the *ISCA1* expression group, the differential expression of chemokine, immune-stimulator, MHC, and receptor genes was investigated (**Figures 4A–E**). The expression was higher in the high expression group, and most of the four different types of genes had substantial variations.

**Figure 5:**
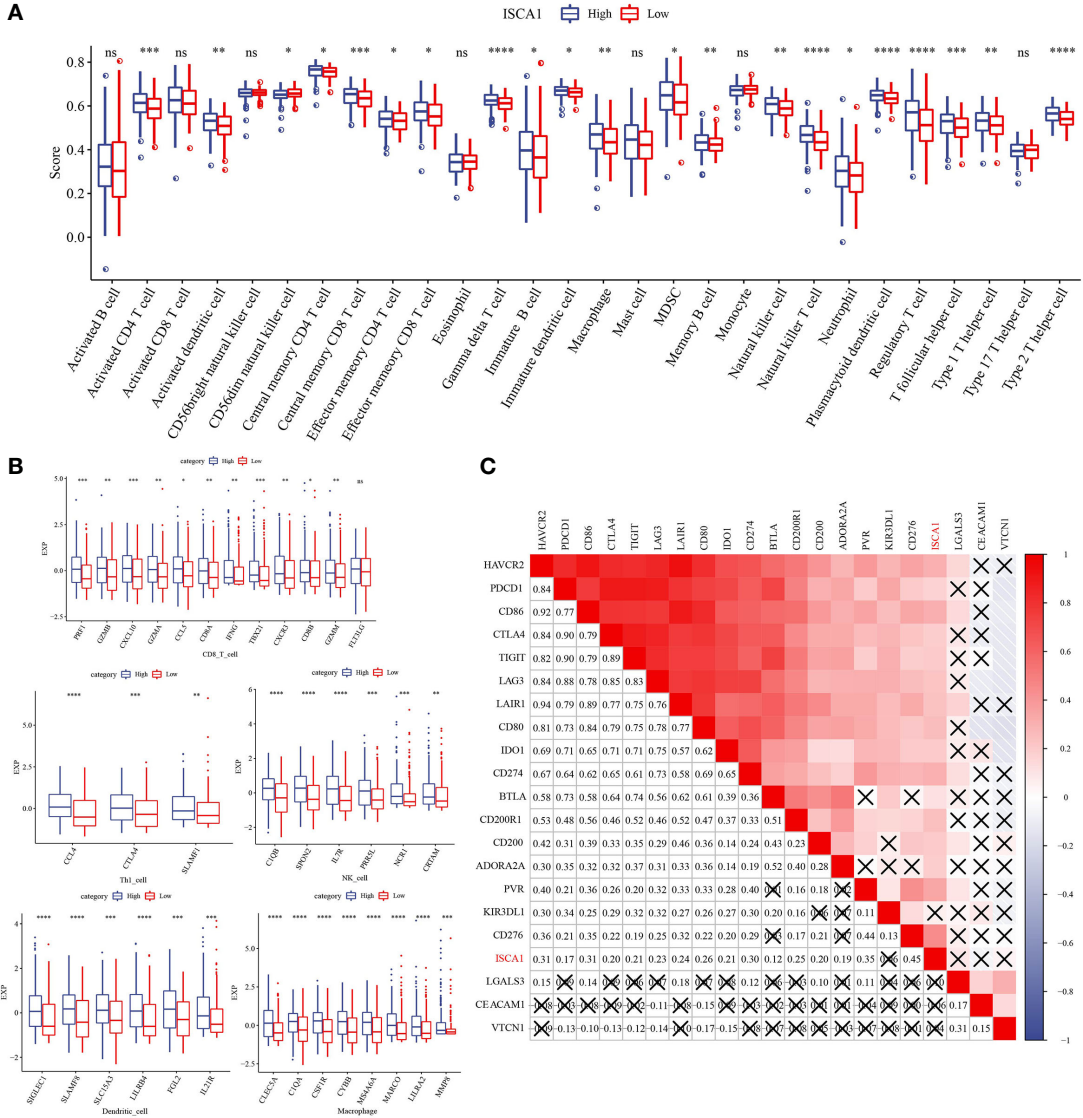
Distribution difference of immune cell score in *ISCA1* group. **(A)** Differences in immune cell score between high *ISCA1* group and low *ISCA1* group. **(B)** Differences in effector genes of five TIICs (cd8+t cells, NK cells, macrophages, Th1 cells, and dendritic cells) related immune cells between the high *ISCA1* group and low *ISCA1* group. **(C)** Correlation between *ISCA1* and immune checkpoints. Color and value represent Spearman correlation coefficient. (X represents p>0.05) (* represents p < 0.05, ** represents p < 0.01, *** represents p < 0.001, **** represents p < 0.0001, ns represents p > 0.05).

The *ISCA1* high expression group had a high immune score, and the distribution differences of 28 immune cell scores in the *ISCA1* group were examined, revealing that 21 had major variations (**Figure 5A**). The immune infiltration of BLCA was next investigated, and a link was observed between *ISCA1* expression and immune infiltration score. The marker genes of five cell types were studied: CD8 T cells, dendritic cells, macrophages, NK cells, and Th1 cells. In the *ISCA1* high expression group, the majority of the genes were highly expressed (**Figure 5B**). The link between *ISCA1* and immunological checkpoints was also measured. Based on the outcomes, *ISCA1* and these immunological checkpoints had a substantial positive link (**Figure 5C**).

### *ISCA1* prediction of clinical response and excessive progression of immune checkpoint blockade in BLCA

**Figure 6:**
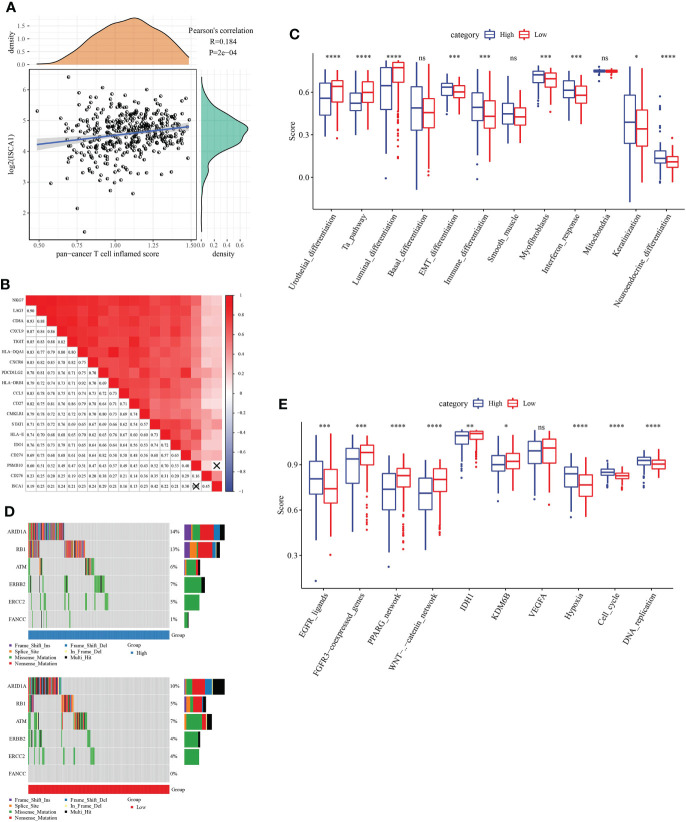
The immune characteristic analysis of ISCA. **(A, B)** Correlation between *ISCA1* and pan-cancer T cell inflammation score, and correlation between individual genes contained in T cell inflammation characteristics. The score of T cell inflammation was positively correlated with the clinical response to cancer immunotherapy. **(C)** Correlation between *ISCA1* and molecular subtypes and BLCA characteristics using seven different algorithms. **(D)** Mutation spectrum of neoadjuvant chemotherapy-related genes in low *ISCA1* group and high *ISCA1* group. **(E)** Correlation between *ISCA1* and enrichment scores of several therapeutic features such as targeted therapy and radiotherapy. *P<0.05, **P<0.01, ***P<0.001, ****P<0.0001, ns>0.05.

The link between *ISCA1* expression value and pan-cancer T cell inflamed score was measured, and the outcomes indicated a major positive link (**Figure 6A**). Moreover, the link between *ISCA1* and different immune properties (immune checkpoint, expression of immunomodulator and TIIC effector genes, and characteristics linked with immunotherapy) in subgroups with varied immune checkpoint blockade (ICB) responses were analyzed (**Figure 6B**). The outcomes suggested that *ISCA1* had a major positive link with them.

By comparing the scores of BLCA tumors and immune-related pathways, it was discovered that there were major variations in related immune pathways in the high *ISCA1* group and the low *ISCA1* group of BLCA tumors, the correlation between *ISCA1* expression and Neuroendocrine differentiation pathway is positive in low *ISCA1* group (**Figure 6C**). For example, the Neuroendocrine_ differentiation pathway score was higher in the high *ISCA1* group, whereas the score was lower in the low *ISCA1* group. ARID1A, RB1, ERBB2, FANCC, and other genes that could be linked to radiotherapy and chemotherapy were compared. It was observed that the mutation frequencies in the high and low *ISCA1* groups were different (**Figure 6D**). ARID1A, RB1, ERBB2, ERCC2, and FANCC mutation frequencies were greater in high *ISCA1* groups, for instance, missense mutation of ERCC2 was 5% in the high *ISCA1* group and 4% in the low *ISCA1* group. In high and low *ISCA1* expression groups, the differences in three categories (EGFR network, immune inhibit oncogenic pathways, and radiotherapy predicted pathways) were compared (**Figure 6E**). The high *ISCA1* group was mostly positively correlated in the EGFR network in the EGFR_ligands pathway, while the low *ISCA1* group was mostly negatively linked.

### Identification of immune-related differential genes and PPI analysis

**Figure 7:**
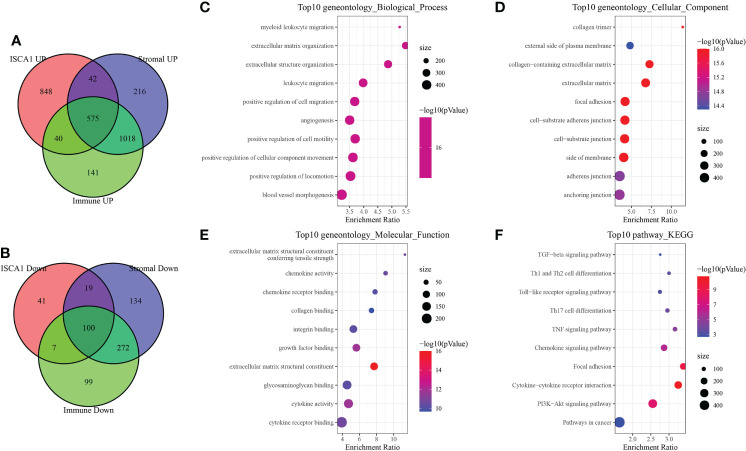
Immune-related differential gene analysis. **(A)** The intersection of up-regulated genes in *ISCA1*, StromalScore, and ImmuneScore. **(B)** Intersection of down-regulated genes in *ISCA1*, StromalScore, and ImmuneScore. **(C–F)** GO and KEGG enrichment analysis of differentially expressed genes in *ISCA1*, StromalScore, and ImmuneScore.

A total of 575 up-regulated genes and 100 down-regulated genes were obtained by grouping the up-regulated and down-regulated genes of the BLCA sample species *ISCA1*, StromalScore, and ImmuneScore, respectively (**Figures 7A, B**). The GO and KEGG function enrichment of differential genes was then analyzed using WebGestaltR. Genes were discovered to be closely linked to cancer and immune pathways such as myeloid leukocyte migration, leukocyte migration, angiogenesis, Th1 and Th2 cell differentiation, and so on (**Figures 7C–F**).

**Figure 8:**
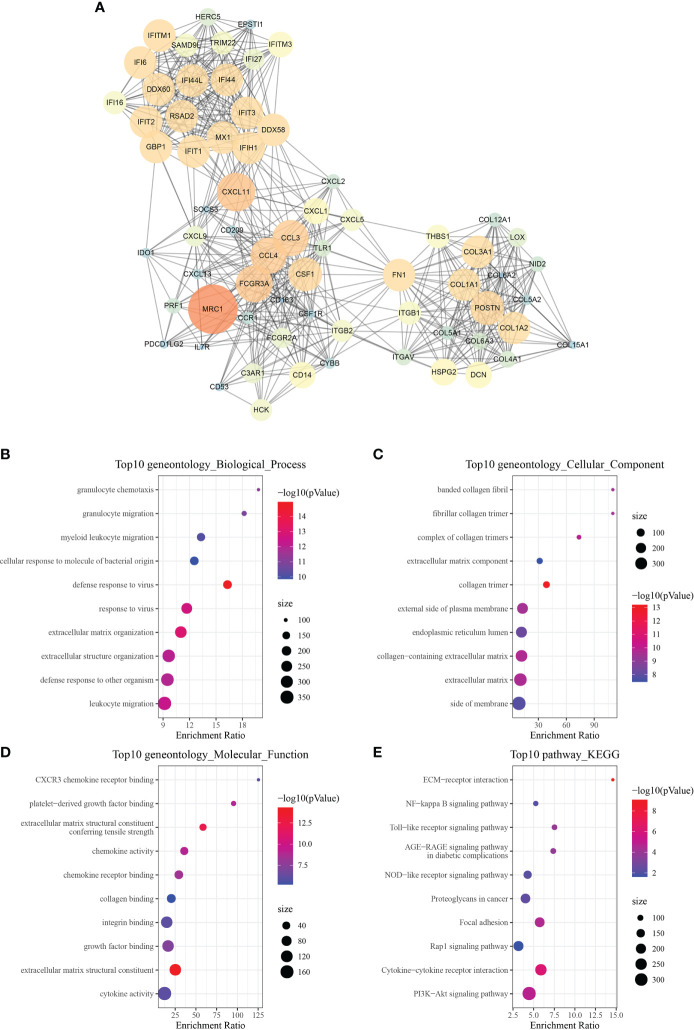
Module function analysis. **(A)** PPI analysis diagram of module Mcode1 (the larger the circle is, the darker the color is, the greater the score of mcodes representing genes is, and the more important the genes are). **(B–E)** GO and KEGG functional enrichment analysis of the gene of module Mcode1.

Using the string website, PPI found and analyzed a total of 675 differential genes. Following that, Cytoscape was used to visualize the data and the MCODE plug-in was utilized to identify significant clusters. There were three gene clusters with more than ten genes each (Mcode1, Mcode2, and Mcode3 respectively). The genes MRC1, CXCL11, CCL3, CCL4, CSF1, and FN1 were all found in Mcode1 (**Figure 8A**). Then, to determine their functions, WebGestaltR was utilized to do a GO and KEGG function enrichment analysis (**Figures 8B–E**). The findings revealed that the Mcode1 module was linked to immunological pathways such as the Toll-like receptor signaling pathway and the interaction between cytokine and cytokine receptors.

### Construction of BLCA cancer immune risk score model

**Figure 9:**
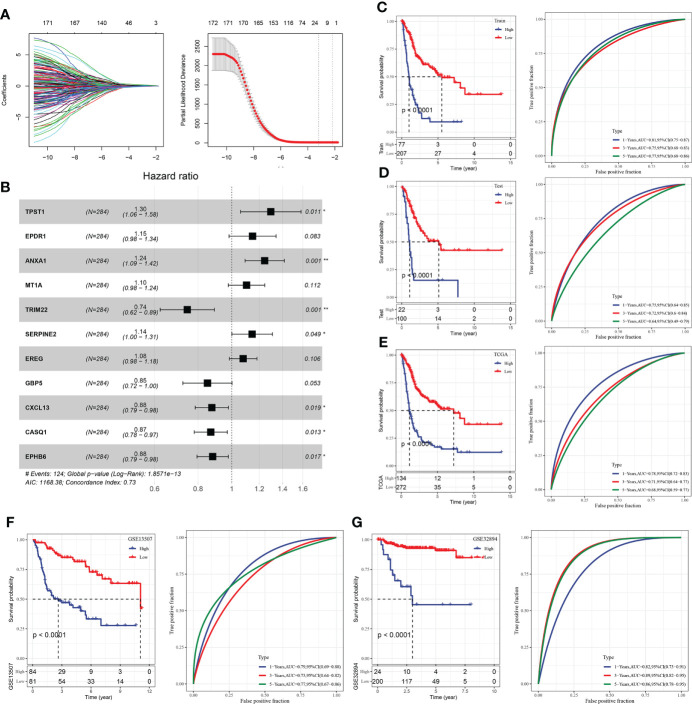
Construction of IRS model of BLCA. **(A)** Lasso coefficient distribution of 40 prognostic RNAs in the GEO training cohort. According to the logarithm (λ) sequence plotting coefficient profile. **(B)** Multifactor results of genes in the final IRS model; **(C)** KM and ROC analysis of IRS model on GEO training dataset. **(D)** KM and ROC analysis of IRS model on GEO validation dataset. **(E)** KM and ROC analysis of IRS model on all GEO datasets. **(F)** KM and ROC analysis of IRS model on all TCGA datasets. **(G)** KM and ROC analysis of IRS model on all ICGC datasets. *P<0.05, **P<0.01.

After the above analysis, 675 we identified the differential genes linked with immunity, and then 172 genes linked with prognosis were provided by univariate analysis (p < 0.05). Afterward, Lasso was employed for selecting the most suited gene for developing the IRS model. Based on the minimum lambda = 0.04090851, we obtained 21 genes (**Figure 9A**). These genes were used for the multivariate analysis. To further decrease gene number, the stepAIC approach was employed. Finally, we got 11 genes (**Figure 9B**), and the risk coefficients of linked genes were obtained. The risk scores of each sample in the training and validation datasets were measured, and the best cutoff score was used to categorize them into high and low-risk groups, with their KM curves and ROC curves demonstrated separately. In the training set, the AUC value for the 1-year survival rate was 0.81, the AUC value for the 3-year survival rate was 0.75, and the AUC value for the 5-year survival rate was 0.77, whereas in the test set, the AUC value for the 1-year survival rate was 0.75, the AUC value for the 3-year survival rate was 0.72, and the AUC value for the 5-year survival rate was 0.64. (**Figures 9C, D**). A greater survival rate (p < 0.0001) was observed in the low-risk group in both the training and validation sets. In addition, all TCGA datasets, GSE13507 datasets, and GSE32894 datasets were used to validate our IRS model (**Figure 9E–G**). The aforesaid datasets’ BLCA tumor samples could also be sorted into high- and low-risk groups with differing prognoses (p < 0.0001), with the low-risk group having a greater rate of survival.

### Performance comparison between IRS and TIDE

**Figure 10:**
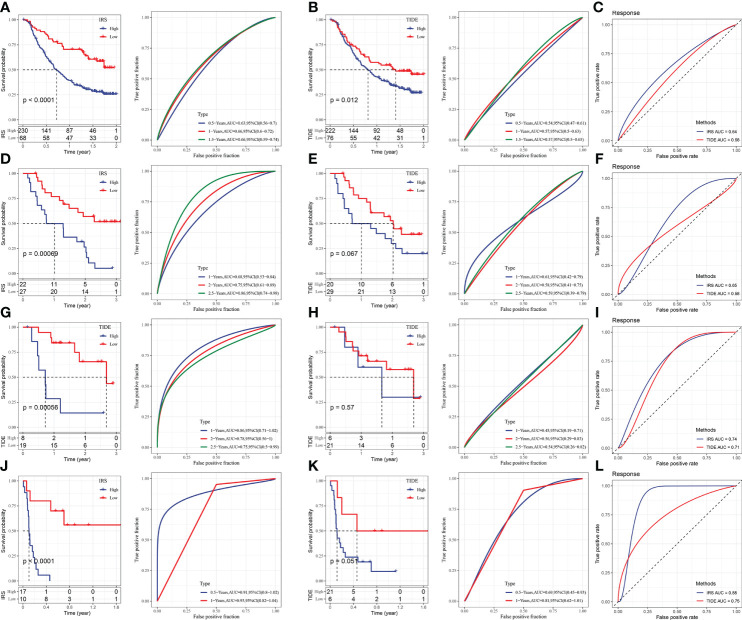
Comparison and analysis of IRS and TIDE. **(A)** IRS survival curve and ROC curve of IMvigor210 dataset. **(B)** TIDE survival curve and ROC curve of IMvigor210 dataset. **(C)** ROC curve of IRS and TIDE on immunotherapy effect in imvigor210 dataset. **(D)** IRS survival curve and ROC curve of GSE91061 dataset. **(E)** TIDE survival curve and ROC curve of GSE91061 dataset. **(F)** ROC curve of IRS and TIDE on immunotherapy effect in GSE91061 dataset. **(G)** IRS survival curve and ROC curve of GSE78220 dataset. **(H)** TIDE survival curve and ROC curve of GSE78220 dataset. **(I)** ROC curve of IRS and TIDE on immunotherapy effect in GSE78220 dataset. **(J)** IRS survival curve and ROC curve of GSE135222 dataset. **(K)** TIDE survival curve and ROC curve of GSE135222 dataset. **(L)** ROC curve of IRS and TIDE for immunotherapy effect in GSE135222 dataset.

The immunotherapy datasets IMvigor210, GSE91061, GSE78220, and GSE135222 were chosen to predict, evaluate, and compare the efficacy scores of immune therapy. Our approach was used to calculate IRS in these data, and TIDE was utilized to evaluate the effect of immunotherapy, after which the predictive effect of IRS and TIDE on treatment response was evaluated. The immunotherapy samples were separated into high and low-scoring groups following the best IRS and TIDE cut-off scores. Our IRS score was higher than the TIDE score, according to the results (**Figure 10**).

## Discussion

Research has suggested that the *ISCA1* gene is downregulated in 11 types of cancer and upregulated in 4 cancer types. Specifically, the expression of *ISCA1* in BLCA was positively linked with the immune score. Therefore, BLCA is the major type of cancer for follow-up analysis and research. BLCA is a highly malignant tumor in the urinary tract. In 2018, there were nearly 549000 new cases and 200000 deaths, ranking the 10th (1). Non-muscle invasive bladder cancer (NMIBC) and muscle-invasive bladder cancer (MIBC) are the two main subtypes of heterogeneous carcinoma (MIBC). The main component of BLCA in NMIBC. It is prone to recur, despite the fact that it is not lethal (26). To prevent recurrence and progression, chemotherapeutic medicines and the BCG vaccine are administered intrathecally (27). Tumor immunology has been the subject of increasing research recently. Many immune checkpoint inhibitors that have been discovered and demonstrated to produce strong and long-lasting responses in cancer patients (28–30). This is consistent with the findings of this study, demonstrating its validity. CTLA4, PDCD1, CD86, and CD274 had strong positive correlations with *ISCA1* in BLCA.

Based on the clinical trials of immune checkpoint inhibitors, the *in situ* infiltration of TME immune cells is now considered important for the prognosis prediction of different cancer types and observation of how they react to immunotherapy (31, 32). As a result, the overall status of TME immune cell infiltration was thoroughly examined by evaluating the distribution difference of 28 immune cell scores in BLCA in the *ISCA1* group. The results revealed that the *ISCA1* group had significantly distinct immune cells, with the group with high *ISCA1* expression having a higher immunological score. Furthermore, because macrophages are immunosuppressive cells, most of their hallmark genes were significantly expressed in the *ISCA1* high expression group. The CD8+T and natural killer cells’ activation was suppressed by these immunosuppressive cells (33). Immunosuppressive cells respond to changes in other immune cells and play a key role in the tumor immunological microenvironment. Therefore, we concluded that the poor prognosis of high expression of *ISCA1* can be linked to this tumor immunosuppressive microenvironment. Moreover, CTLA-4, PD-1/PD-L1, and other immune checkpoints also functioned as rheostats in regulating the immune response by preventing the initiation and immune monitoring of protective immune cells (34, 35). We observed that the expression of immune checkpoints was greatly enhanced in the high expression group of *ISCA1*, which suggests that *ISCA1* might be helpful in predicting the effect of immune checkpoint inhibitor therapy. *ISCA1* was found to be useful in immunotherapy response prediction in the TCGA-BLCA cohort using the IRS model and TIDE algorithm. All of this suggested that *ISCA1* was a useful biomarker for the immunotherapy response prediction.

However, even if the data from various databases were studied and integrated, the current report still has certain limitations. First, while bioinformatics analysis supplied us with some useful information on *ISCA1*’s role in cancer, we still needed *in vitro* or *in vivo* biology experiments to confirm our findings and boost therapeutic use. More research on the mechanism of *ISCA1*’s function at the molecular and cellular levels would be beneficial. Second, although post-translational modification was important in controlling intracellular signal transduction and regulatory factor activity, no post-translational modification information for *ISCA1* was found in these databases. Furthermore, whereas *ISCA1* expression was linked to both immunological and clinical survival in human cancer, it was unclear whether *ISCA1* affected clinical survival *via* the immune pathway.

Finally, the first pan-cancer investigation of *ISCA1* indicated that the factor was differently expressed between tumor and normal tissues, as well as a link between *ISCA1* expression and BLCA clinical outcome. Our outcomes show that the level of *ISCA1* expression influences prognosis. Further research into the involvement of *ISCA1* in each cancer is required. *ISCA1* expression in BLCA is also linked to the invasion of different immune cells. These outcomes may help in clarifying the role of *ISCA1* in tumorigenesis and development, particularly in BLCA, and give a reference for more accurate and tailored immunotherapy in the future.

## Conclusion

Overall, our outcomes indicated that *ISCA1* is involved in the progression of pan-cancer, particularly in BLCA. In BLCA, the high expression of *ISCA1* predicted a worse prognosis, and the immune scores of some immune cells indicated a major positive link with them. Finally, an IRS model was developed, and the *ISCA1*-related low-risk group had a higher survival rate. In conclusion, the possibility of *ISCA1* as a biomarker for predicting pan-cancer was evaluated comprehensively, and its value in BLCA was determined, which expanded our vision in immunotherapy and can provide a useful evaluation system for clinical application.

## Data availability statement

The original contributions presented in the study are included in the article/**Supplementary Material**. Further inquiries can be directed to the corresponding author.

## Author contribution

All authors contributed to this present work: RLZ designed the study, NXP acquired the data. WL drafted the manuscript and revised the manuscript. All authors read and approved the manuscript.

## Conflict of interest

The authors declare that the research was conducted in the absence of any commercial or financial relationships that could be construed as a potential conflict of interest.

## Publisher’s note

All claims expressed in this article are solely those of the authors and do not necessarily represent those of their affiliated organizations, or those of the publisher, the editors and the reviewers. Any product that may be evaluated in this article, or claim that may be made by its manufacturer, is not guaranteed or endorsed by the publisher.

## References

[1] Bray F Ferlay J Soerjomataram I Siegel RL Torre LA Jemal A. Global cancer statistics 2018: GLOBOCAN estimates of incidence and mortality worldwide for 36 cancers in 185 countries. CA: Cancer J Clin (2018) 68(6):394–424. doi: 10.3322/caac.21492 30207593

[2] Ribas A Wolchok JD. Cancer immunotherapy using checkpoint blockade. Sci (New York NY) (2018) 359(6382):1350–5. doi: 10.1126/science.aar4060 PMC7391259 29567705

[3] Blum A Wang P Zenklusen JC. SnapShot: TCGA-analyzed tumors. Cell (2018) 173(2):530. doi: 10.1016/j.cell.2018.03.059 29625059

[4] Roth KG Mambetsariev I Kulkarni P Salgia R. The mitochondrion as an emerging therapeutic target in cancer. Trends Mol Med (2020) 26(1):119–34. doi: 10.1016/j.molmed.2019.06.009 PMC6938552 31327706

[5] Jia D Park JH Jung KH Levine H Kaipparettu BA. Elucidating the metabolic plasticity of cancer: Mitochondrial reprogramming and hybrid metabolic states. Cells (2018) 7(3):21. doi: 10.3390/cells7030021 PMC5870353 29534029

[6] Sonveaux P Végran F Schroeder T Wergin MC Verrax J Rabbani ZN. Targeting lactate-fueled respiration selectively kills hypoxic tumor cells in mice. J Clin Invest (2008) 118(12):3930–42. doi: 10.1172/JCI36843 PMC2582933 19033663

[7] Arcucci A Ruocco MR Granato G Sacco AM Montagnani S. Cancer: An oxidative crosstalk between solid tumor cells and cancer associated fibroblasts. BioMed Res Int (2016) 2016:4502846. doi: 10.1155/2016/4502846 27595103 PMC4993917

[8] Yang X Lu D Zhang X Chen W Gao S Dong W. Knockout of ISCA1 causes early embryonic death in rats. Anim Models Exp Med (2019) 2(1):18–24. doi: 10.1002/ame2.12059 PMC6431120 31016283

[9] Sheftel AD Wilbrecht C Stehling O Niggemeyer B Elsässer HP Mühlenhoff U. The human mitochondrial ISCA1, ISCA2, and IBA57 proteins are required for [4Fe-4S] protein maturation. Mol Biol Cell (2012) 23(7):1157–66. doi: 10.1091/mbc.e11-09-0772 PMC3315811 22323289

[10] Banci L Brancaccio D Ciofi-Baffoni S Del Conte R Gadepalli R Mikolajczyk M. [2Fe-2S] cluster transfer in iron-sulfur protein biogenesis. Proc Natl Acad Sci United States Am (2014) 111(17):6203–8. doi: 10.1073/pnas.1400102111 PMC4035983 24733926

[11] Kovacheva M Zepp M Berger S Berger MR. Conditional knockdown of integrin beta-3 reveals its involvement in osteolytic and soft tissue lesions of breast cancer skeletal metastasis. J Cancer Res Clin Oncol (2021) 147(2):361–71. doi: 10.1007/s00432-020-03428-y PMC7817553 33083904

[12] Barbie DA Tamayo P Boehm JS Kim SY Moody SE Dunn IF. Systematic RNA interference reveals that oncogenic KRAS-driven cancers require TBK1. Nature (2009) 462(7269):108–12. doi: 10.1038/nature08460 PMC2783335 19847166

[13] Yoshihara K Shahmoradgoli M Martínez E Vegesna R Kim H Torres-Garcia W. Inferring tumour purity and stromal and immune cell admixture from expression data. Nat Commun (2013) 4:2612. doi: 10.1038/ncomms3612 24113773 PMC3826632

[14] Chen B Khodadoust MS Liu CL Newman AM Alizadeh AA. Profiling tumor infiltrating immune cells with CIBERSORT. Methods Mol Biol (Clifton NJ) (2018) 1711:243–59. doi: 10.1007/978-1-4939-7493-1_12 PMC5895181 29344893

[15] Li T Fu J Zeng Z Cohen D Li J Chen Q. TIMER2.0 for analysis of tumor-infiltrating immune cells. Nucleic Acids Res (2020) 48(W1):W509–w14. doi: 10.1093/nar/gkaa407 PMC7319575 32442275

[16] Becht E Giraldo NA Lacroix L Buttard B Elarouci N Petitprez F. Estimating the population abundance of tissue-infiltrating immune and stromal cell populations using gene expression. Genome Biol (2016) 17(1):218. doi: 10.1186/s13059-016-1070-5 27765066 PMC5073889

[17] Plattner C Finotello F Rieder D. Deconvoluting tumor-infiltrating immune cells from RNA-seq data using quanTIseq. Methods Enzymol (2020) 636:261–85. doi: 10.1016/bs.mie.2019.05.056 32178821

[18] Aran D Hu Z Butte AJ. xCell: digitally portraying the tissue cellular heterogeneity landscape. Genome Biol (2017) 18(1):220. doi: 10.1186/s13059-017-1349-1 29141660 PMC5688663

[19] Ritchie ME Phipson B Wu D Hu Y Law CW Shi W. Limma powers differential expression analyses for RNA-sequencing and microarray studies. Nucleic Acids Res (2015) 43(7):e47. doi: 10.1093/nar/gkv007 25605792 PMC4402510

[20] Liao Y Wang J Jaehnig EJ Shi Z Zhang B. WebGestalt 2019: gene set analysis toolkit with revamped UIs and APIs. Nucleic Acids Res (2019) 47(W1):W199–w205. doi: 10.1093/nar/gkz401 31114916 PMC6602449

[21] Friedman J Hastie T Tibshirani R. Regularization paths for generalized linear models *via* coordinate descent. J Stat Software (2010) 33(1):1–22. doi: 10.18637/jss.v033.i01 PMC2929880 20808728

[22] Zhang Z. Variable selection with stepwise and best subset approaches. Ann Trans Med (2016) 4(7):136. doi: 10.21037/atm.2016.03.35 PMC4842399 27162786

[23] Jiang P Gu S Pan D Fu J Sahu A Hu X. Signatures of T cell dysfunction and exclusion predict cancer immunotherapy response. Nat Med (2018) 24(10):1550–8. doi: 10.1038/s41591-018-0136-1 PMC6487502 30127393

[24] Sato M Kobayashi M Wakamatsu Y Imai M Horibe M. [Symposium–drug action and animal behavior. 7. pharmacological studies of emotional behavior of rodentia]. Nihon Yakurigaku Zasshi Folia Pharmacol Japonica (1970) 66(4):80–1. 5531419

[25] Ru B Wong CN Tong Y Zhong JY Zhong SSW Wu WC. TISIDB: an integrated repository portal for tumor-immune system interactions. Bioinf (Oxford England) (2019) 35(20):4200–2. doi: 10.1093/bioinformatics/btz210 30903160

[26] Babjuk M Böhle A Burger M Capoun O Cohen D Compérat EM. EAU guidelines on non-muscle-invasive urothelial carcinoma of the bladder: Update 2016. Eur Urol (2017) 71(3):447–61. doi: 10.1016/j.eururo.2016.05.041 27324428

[27] Soria F Milla P Fiorito C Pisano F Sogni F Di Marco M. Efficacy and safety of a new device for intravesical thermochemotherapy in non-grade 3 BCG recurrent NMIBC: a phase I-II study. World J Urol (2016) 34(2):189–95. doi: 10.1007/s00345-015-1595-3 26026818

[28] Atkins MB Clark JI Quinn DI. Immune checkpoint inhibitors in advanced renal cell carcinoma: experience to date and future directions. Ann Oncol (2017) 28(7):1484–94. doi: 10.1093/annonc/mdx151 28383639

[29] Johnson DB Sullivan RJ Menzies AM. Immune checkpoint inhibitors in challenging populations. Cancer (2017) 123(11):1904–11. doi: 10.1002/cncr.30642 PMC5445005 28241095

[30] Inman BA Longo TA Ramalingam S Harrison MR. Atezolizumab: a pd-l1-blocking antibody for bladder cancer. Clin Cancer Res (2017) 23(8):1886–90. doi: 10.1158/1078-0432.CCR-16-1417 27903674

[31] Hegde PS Karanikas V Evers S. The where, the when, and the how of immune monitoring for cancer immunotherapies in the era of checkpoint inhibition. Clin Cancer Res (2016) 22(8):1865–74. doi: 10.1158/1078-0432.CCR-15-1507 27084740

[32] Lee JM Lee MH Garon E Goldman JW Salehi-Rad R Baratelli FE. Phase I trial of intratumoral injection of ccl21 gene-modified dendritic cells in lung cancer elicits tumor-specific immune responses and cd8(+) t-cell infiltration. Clin Cancer Res (2017) 23(16):4556–68. doi: 10.1158/1078-0432.CCR-16-2821 PMC5599263 28468947

[33] Bunt SK Yang L Sinha P Clements VK Leips J Ostrand-Rosenberg S. Reduced inflammation in the tumor microenvironment delays the accumulation of myeloid-derived suppressor cells and limits tumor progression. Cancer Res (2007) 67(20):10019–26. doi: 10.1158/0008-5472.CAN-07-2354 PMC4402704 17942936

[34] Le Mercier I Lines JL Noelle RJ. Beyond ctla-4 and pd-1, the generation z of negative checkpoint regulators. Front Immunol (2015) 6:418. doi: 10.3389/fimmu.2015.00418 26347741 PMC4544156

[35] Dempke WCM Fenchel K Uciechowski P Dale SP. Second- and third-generation drugs for immuno-oncology treatment-the more the better? Eur J Cancer (Oxford England: 1990) (2017) 74:55–72. doi: 10.1016/j.ejca.2017.01.001 28335888

