# Supplementary figures and images for: Identification of ISCA1 as novel immunological and prognostic biomarker for bladder cancer
Source: Front Immunol. 2022 Aug 22;13:975503. doi: 10.3389/fimmu.2022.975503 (PMC9442282; doi:10.3389/fimmu.2022.975503)

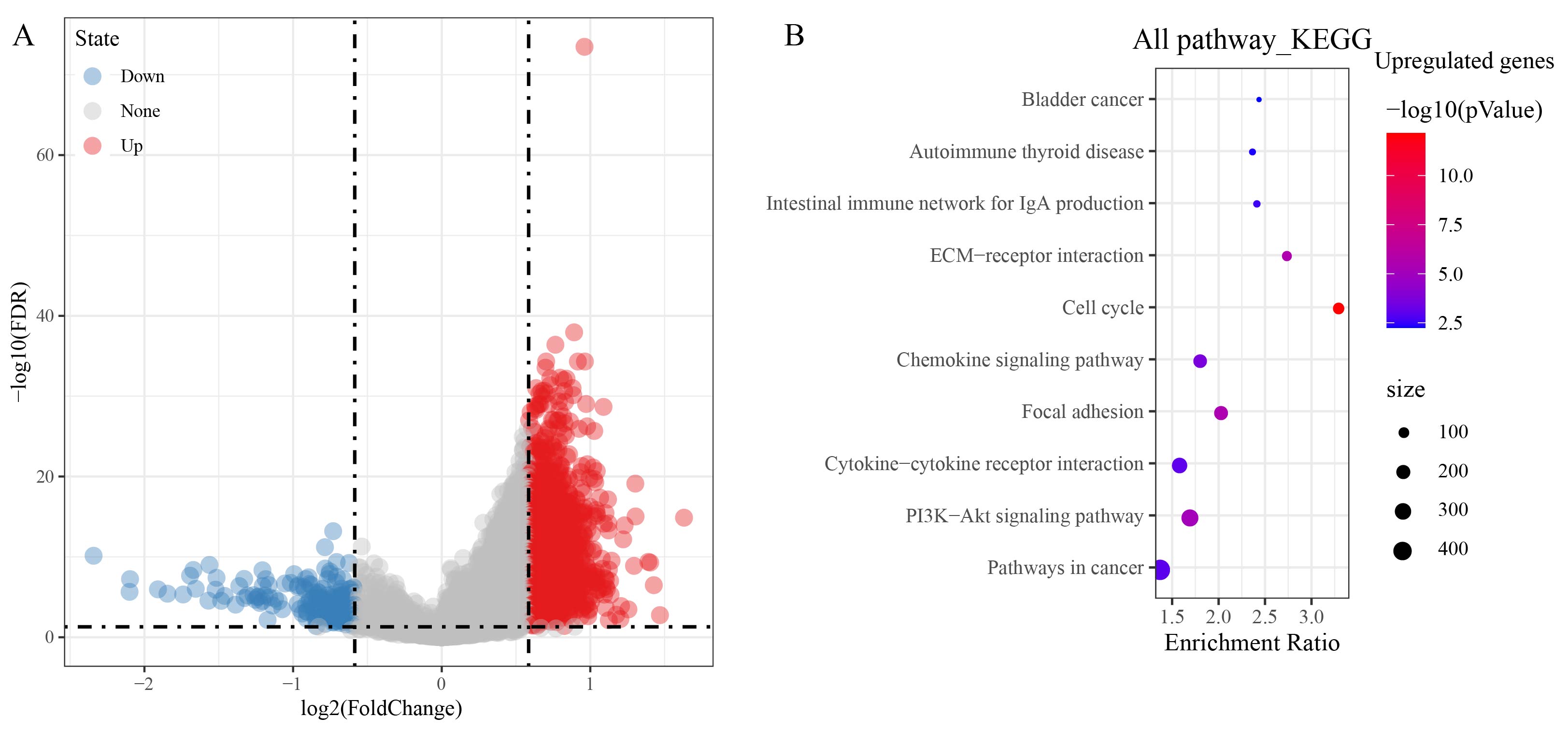

Supplement: Supplementary file 1 [file Image_1.jpeg]
